# One-Stage Laparoscopic Surgery for Pulmonary Sequestration and Hiatal Hernia in a 2-Year-Old Girl

**DOI:** 10.1055/s-0037-1612611

**Published:** 2018-01-08

**Authors:** Hisayuki Miyagi, Shohei Honda, Hiromi Hamada, Masashi Minato, Momoko W. Ara, Akinobu Taketomi

**Affiliations:** 1 Department of Gastroenterological Surgery I, Hokkaido University Graduate School of Medicine, Kita-ku, Kita 15, Nishi 7, Sapporo, Japan; 2 Department of Pediatric Surgery, Hokkaido Medical Center for Child Health and Rehabilitation, Teine-ku, Kanayama 1-1, Sapporo, Japan

**Keywords:** pulmonary sequestration, hiatal hernia, gastroesophageal reflux, laparoscopic, one-stage

## Abstract

We herein report a case of one-stage laparoscopic surgery for extralobar pulmonary sequestration (EPS) and hiatal hernia. Our patient was a 2-year-old girl who was diagnosed as a mediastinal mass lesion. Postnatal computed tomography revealed that the mediastinal mass was an EPS. Two weeks after birth, the patient developed gastroesophageal reflux (GER), and esophagography showed a hiatal hernia. At 2 years of age, she underwent one-stage laparoscopic Nissen's fundoplication for GER with resection of the EPS in the posterior mediastinum. The sequestrated lung was grasped via the esophageal hiatus; three aberrant blood vessels were dissected to allow removal of the sequestration through the umbilical port site. The esophageal hiatus was repaired and Nissen's fundoplication was performed laparoscopically. The patient's postoperative course was uneventful, with no recurrence of GER symptoms for 1 year. We conclude that one-stage laparoscopic surgery is useful for patients with EPS and hiatal hernia.

## Introduction

Although extralobar pulmonary sequestration (EPS) is a rare congenital lung anomaly, it is the second most common lung malformation in children, accounting for 0.1 to 1.8% of cases. 1 The origin of the condition remains uncertain; however, the available evidence suggests that it results from accessory lung tissue arising from the foregut. 1 2 EPS is located in the mediastinum in approximately 5% of cases. 2 We herein report the first case of one-stage laparoscopic surgery for an EPS with a hiatal hernia.

## Case Report

**Fig. 1:**
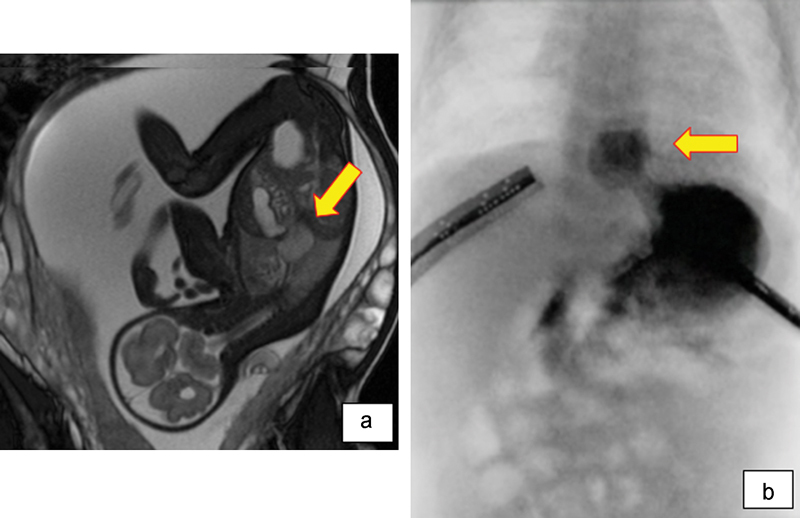
(a) Fetal magnetic resonance imaging shows a cystic lesion with slightly high intensity in true FISP and HASTE mode (arrow). (b) Esophagography shows a hiatal hernia and gastroesophageal reflux (arrow).

**Fig. 2:**
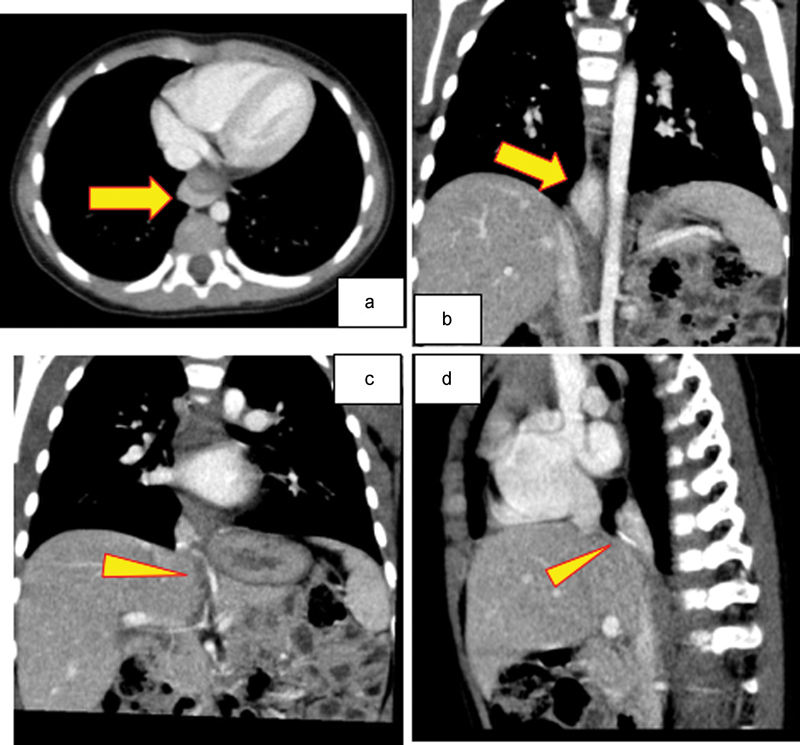
(a, b) Postnatal computed tomography shows two anomalous systemic arteries originating from the left gastric artery and supplying the lung mass. (c, d) All drainage veins in the mass drained to the portal vein.

**Fig. 3:**
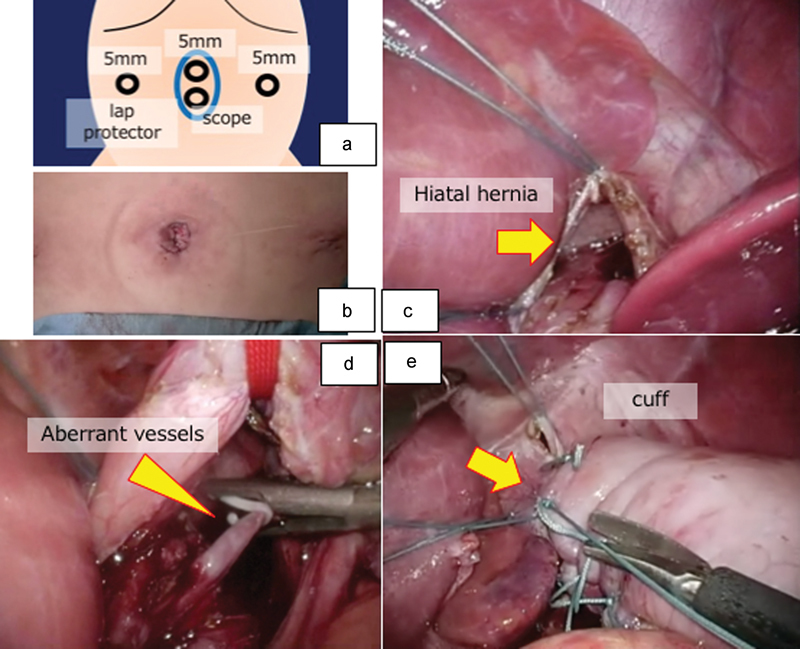
(a, b) Three port sites were established. (c) To secure the surgical field, we used stay sutures in the hernia ring to expand this field and elevate the left liver lobe. We elevated the esophagus to prepare for fundoplication in the usual manner. (d) After dissection of three aberrant vessels, the sequestrated lung was grasped via the esophageal hiatus and removed through the umbilical port site. (e) The esophageal hiatus was repaired and Nissen's fundoplication was performed in the usual manner.

A 2-year-old girl was diagnosed with fetal magnetic resonance imaging as a mediastinal mass lesion ( Fig. 1a ). She was delivered by cesarean section at 32 weeks' gestation with a birth weight of 1,890 g. EPS was diagnosed with postnatal computed tomography (CT). The CT at 2 years of age showed two anomalous systemic arteries originating from the left gastric artery and supplying the lung mass. All drainage veins in the mass drained to the portal vein ( Fig. 2a – d ). Two weeks after birth, the patient developed gastroesophageal reflux (GER), and esophagography showed a hiatal hernia ( Fig. 1b ). She was initially treated by mosapride, which is a gastroprokinetic agent that acts as a selective 5-hydroxytryptamine receptor 4 agonist. At 2 years of age (body weight of 10.7 kg and height of 76.5 cm, both within ± 2.0 standard deviations), the patient underwent one-stage laparoscopic Nissen's fundoplication for treatment of the GER and resection of the EPS in the posterior mediastinum because she sometimes experienced nausea and we considered that the EPS might be associated with this symptom. Three port sites were used ( Fig. 3a ), and a wound edge protector was inserted at the umbilical site to allow use of the scope and grasping forceps. To better expose the surgical field, we used stay sutures (3–0 ETHIBOND; Ethicon, Somerville, New Jersey, United States) in the hernia ring to expand this field and elevate the left liver lobe ( Fig. 3c ). We elevated the esophagus to prepare for fundoplication in the usual manner. After dissection of three aberrant vessels, the sequestrated lung was grasped via the esophageal hiatus and removed through the umbilical port site. We used size medium polymer ligating clips (Hem-o-lok; Teleflex, Wayne, Pennsylvania, United States) that were entered through the 5-mm port for aberrant vascular control to avoid burn injury from hemoclips when using energy devices intraoperatively ( Fig. 3d ). The anterior and posterior aspects of the esophageal hiatus were repaired with ETHIBOND sutures, and Nissen's fundoplication was performed in the usual manner ( Fig. 3e ). This procedure was performed with only three port sites ( Fig. 3b ). The pathological diagnosis was EPS. The patient's postoperative course was uneventful, with no recurrence of GER symptoms after 1 year of follow-up.

## Discussion

A laparoscopic approach allows extensive exploration of the mediastinum and abdominal cavity through small incisions, and magnification allows good control of systemic vessels. In this case, we performed one-stage laparoscopic surgery for an EPS with hiatal hernia because two-stage surgery is often associated with a longer operative time, greater blood loss due to adhesion, and more frequent complications such as esophageal perforation and pneumothorax. 3 4 Our patient's postoperative course was uneventful; thus, we conclude that the risks of surgical morbidity in this case were lower than the risks of long-term complications of the sequestration itself, such as recurrent infections. 1

In the present case, the patient had GER resulting from a hiatal hernia with EPS. Sequestration with both gastric and intradiaphragmatic components necessitates a global definition of bronchopulmonary foregut malformation, considering sequestration as a complex parenchymal maldevelopment rather than the association of separate lesions. 5 As the natural evolution of both intralobar and extralobar sequestrations is unknown, it is difficult to provide evidence-based arguments for its treatment. Nevertheless, in the case of EPS concomitant with GER, surgical treatment at an early period should be considered to prevent the development of firm adhesion between the esophagus and surrounding sequestration lesion caused by long-term reflux esophagitis.

As with all congenital lung malformations, the natural evolution of both intralobar and extralobar bronchopulmonary sequestrations is unknown, making it difficult to provide evidence-based arguments for treatment. 6 Clinical manifestations of bronchopulmonary sequestration increase over time, leading to respiratory symptoms, local hemorrhage, and recurrent infections. 2 7 8 Aneurysmal evolution of an aberrant systemic artery has also been reported. 9 However, surgery for symptomatic congenital lung malformations is associated with a longer operative time, greater blood loss, a longer hospital stay, and more frequent postoperative complications than surgery for other conditions. 3 4

In conclusion, one-stage laparoscopic surgery is useful for patients with a hiatal hernia and EPS. Resection of the EPS through the hiatus was a reasonable approach for this patient because the EPS was located at the lower posterior mediastinum and the aberrant vessels were near the hiatus. However, this approach might not be suitable for patients in whom the EPS is large or the aberrant vessels are far from the hiatus. We suggest that this approach might be adapted in many other cases of EPS with GER.

## New Insights and the Importance for the Pediatric Surgeon

One-stage laparoscopic surgery is useful for patients with hiatal hernia and EPS, especially when the EPS is located in the posterior mediastinum near the diaphragm. This approach is a single-stage treatment that reduces the risk of postoperative GER and thoracic deformity because of the laparoscopic approach; however, the best treatment choice for any patient with EPS and hiatal hernia must be limited to the location of the EPS.

## References

[1] Chouikh T Berteloot L Revillon Y Delacourt C Khen-Dunlop N Extralobar pulmonary sequestration with combined gastric and intradiaphragmatic locations Pediatr Pulmonol 2014 49 05 512 514 24022880 10.1002/ppul.22891

[2] Bush A Prenatal presentation and postnatal management of congenital thoracic malformations Early Hum Dev 2009 85 11 679 684 19758773 10.1016/j.earlhumdev.2009.08.056

[3] Seong Y W Kang C H Kim J T Moon H J Park I K Kim Y T Video-assisted thoracoscopic lobectomy in children: safety, efficacy, and risk factors for conversion to thoracotomy Ann Thorac Surg 2013 95 04 1236 1242 23453743 10.1016/j.athoracsur.2013.01.013

[4] Conforti A Aloi I Trucchi A Asymptomatic congenital cystic adenomatoid malformation of the lung: is it time to operate? J Thorac Cardiovasc Surg 2009 138 04 826 830 19660296 10.1016/j.jtcvs.2009.01.014

[5] Langston C New concepts in the pathology of congenital lung malformations Semin Pediatr Surg 2003 12 01 17 37 12520470 10.1053/spsu.2003.00001

[6] Laberge J M Puligandla P Flageole H Asymptomatic congenital lung malformations Semin Pediatr Surg 2005 14 01 16 33 15770585 10.1053/j.sempedsurg.2004.10.022

[7] Berna P Cazes A Bagan P Riquet M Intralobar sequestration in adult patients Interact Cardiovasc Thorac Surg 2011 12 06 970 972 21362733 10.1510/icvts.2010.263897

[8] Cao C Bi M Hendel N Yan T D An unusual presentation of recurrent pneumonia Lancet 2012 379(9811):192 22243825 10.1016/S0140-6736(11)61588-2

[9] Shyam S Sagar A Sureka J Jakkani R K An unusual case of a giant aneurysm of an aberrant systemic artery supplying a pulmonary sequestration Eur J Cardiothorac Surg 2012 42 03 592 22696457 10.1093/ejcts/ezs295

